# New Insights into Pore Structure and Hydraulic Conductivity of Sodium Hydroxide Alkali-Activated Slag through Advanced Modelling

**DOI:** 10.3390/ma17020363

**Published:** 2024-01-11

**Authors:** Marco Sirotti, Jérôme Carette, Stéphanie Staquet

**Affiliations:** BATir Department, Université Libre de Bruxelles, CP194/02, 50 Avenue F.D. Roosevelt, 1050 Brussels, Belgium; jerome.carette@ulb.be (J.C.); stephanie.staquet@ulb.be (S.S.)

**Keywords:** alkali-activated slag, pore size distribution, hydraulic conductivity, pore surface area, porosity

## Abstract

The study of alkali-activated slag (AAS) is motivated by the need for more sustainable alternatives to Portland cement (PC) within the construction industry. Specifically, AAS offers good mechanical and chemical properties. However, the influence of the activator on its pore structure and hydraulic conductivity remains unclear. Both pore structure and hydraulic conductivity are key parameters in understanding the drying process and could potentially explain the high drying shrinkage observed so far. The present study aims to investigate the pore size distribution and hydraulic conductivity of six distinct AAS/sodium hydroxide mortar compositions, with a particular emphasis on the effect of varying the activator’s molarity and the solution-to-binder ratio (s/b). This research uses the mass variation in different relative humidity (RH) conditions from experimental tests to model the pore surface area, the pore size distribution, and the hydraulic conductivity. From the results, it emerges that increasing the molarity from 0.5 to 8 M reduces the open porosity and refines the pore structure, while increasing the s/b from 0.5 to 0.8 increases the open porosity while refining the pore structure. In addition, high molarity compositions are not suitable for testing in high RH and natural carbonation conditions due to the occurrence of deliquescence. Moreover, the main drying mechanism in AAS is water vapour transport even at high relative humidity, contrary to what was observed in the literature for PC. Finally, the hydraulic conductivity of alkali-activated slag presents a minimum of around 85% RH against the 60–70% RH for PC, causing AAS to dry faster when the relative humidity decreases from 85 to 50%.

## 1. Introduction

In the context of reducing CO_2_ emissions worldwide, the use of alkali-activated blast furnace slag instead of Portland cement (PC) would reduce the carbon footprint of the construction industry and would allow the reuse of industrial by-products [1,2,3,4]. Specifically, AAS presents promising mechanical properties and chemical durability [5,6,7,8]. The main issues hindering the wide adoption of said materials, except for the still high costs, are doubts concerning their long-term durability [7,9,10], specifically the high drying shrinkage observed when compared to PC [7,11]. The main mechanisms of drying shrinkage in porous materials are capillary pressure in capillary pores, disjoining pressure, and surface free tension [12,13,14,15]. All of them are water-related and strongly depend on the size of the pores involved in the drying process [16,17]; therefore, investigating the pore structure and the water transport properties of a porous material provides a crucial insight into its behaviour when exposed to drying.

Previous studies show the effects of the activator, its concentration, and its quantity on the drying shrinkage magnitude of alkali-activated slag [5,7,18,19,20]. In addition, the results presented by Ye et al. [19] confirm that increasing the concentration of NaOH reduces the open porosity and provokes a general refinement of the pore structure, in line with what was observed for other alkali-activated materials [7,18,21,22,23,24,25,26]. Nevertheless, the effects of increasing the activator quantity on the pore structure remain unclear. Similarly, there is very limited knowledge on the water permeability of AAS: blast furnace slag activated using sodium silicate and sodium carbonate presents higher water permeability than PC [24]. On the other hand, we have no indications of the main water transport mechanism, of the effect of the activator concentration, nor of whether the conclusions found for sodium silicate and sodium carbonate are true for sodium hydroxide as well.

Water vapour desorption (WVD) in isotherm conditions is a simple and effective methodology for studying the pore structure of a material. Specifically, it is based on the mass variation when the material is exposed to different levels of relative humidity. The advantage of using water vapour instead of other gases such as CO_2_ or N_2_ [27,28,29,30,31,32] is that the water molecules are relatively smaller than the other ones, allowing them to penetrate into smaller pores and avoid the ink bottle effect [27,33]. Moreover, the test can be performed at room temperature and with no need to remove the gas from the sample before the test and risk provoking microstructural damages. The data obtained from WVD curves can then be used to investigate several properties of the material’s pore structure, namely, the surface area of the pores, the pores’ sizes, and the hydraulic conductivity of the material. The pore surface area is generally obtained using the Brunauer–Emmett–Teller (BET) method [30,32,34,35] and represents the specific surface area of a solid material accessible to water vapour. The theory is a derivation of Langmuir’s adsorption model [36], which describes the adsorption of gas molecules on the surface of solid materials in isothermal conditions when varying the gas pressure. Specifically, one of the main assumptions of the model is the formation of a uniform layer of gas molecules on the material. The BET theory extends Langmuir’s adsorption model for multimolecular layers. Moreover, the BET theory implies that the monolayer is a fictional quantity as the surface of the pores is never completely covered by adsorbed gas until the sample reaches saturation [37]. In addition, the computation of the pore size distribution needs some theoretical assumptions, such as that the pores have a cylindrical shape [38]. Regardless of the limitations, the obtained desorption isotherm curves provide quantitative information used to compute the pore size distribution and specific area of the pores.

In addition to the BET surface area, the WVD curves allow us to compute the pore size distribution of the material due to the Barrett–Joyner–Halenda (BJH) method [31,33,38,39]. The BJH method is based on the Kelvin–Laplace equation, which provides a correlation between pore condensation pressure and pore diameter, and Wheeler’s theory [40]. Specifically, the use of the BJH method allows us to use the WVD curves to estimate the volume and area of the pores of the investigated material.

Finally, the WVD curves are also useful for the modelling of the water transport properties of the material [41], without suffering from all the shortcomings of alternative methodologies, such as cracking due to the preliminary drying of the specimens or the dependency on the nonstraightforward determination of calibration curves [41,42,43,44,45]. The investigation of the water transport properties of a porous material is crucial to understanding its drying process, the relative humidity effect on its hydraulic conductivity, and the relevance of the liquid water and water vapour transport contribution to its total hydraulic conductivity.

This paper compares the pore structure and hydraulic conductivity of different AAS compositions obtained from blast furnace slag (BFS) and sodium hydroxide with three levels of molarity and two solution-to-binder ratios in order to understand the effect of molarity and the quantity of activators.

## 2. Experimental Procedure

### 2.1. Materials

The investigated alkali-activated material compositions were obtained by mixing ground granulated blast furnace slag from Ecocem Benelux B.V. in Moerdijk, The Netherlands with different sodium hydroxide solutions. The volume-mean particle size d_50_ of the slag is equal to 11.6 μ m, and its oxide composition is summarised in Table 1.

Concerning the NaOH solution, three different molarities of 0.5, 2, and 8 M were used in order to understand the effect of the activator concentration on the final properties of the material. In addition, two solution-to-binder ratios of 0.5 and 0.8 were studied to consider the effect of the solution content on the material. Table 2 summarises the six different compositions used in the study. Finally, all tests were carried out at the mortar scale with a sand-to-paste ratio in mass of 1:1, where the paste considers both the precursor and the activating solution. The choice of reducing the sand content, compared to cement standards, was due to the impossibility of mixing high molarity compositions. The sand was also oven-dried at 105 °C for 24 h and stored in airtight containers for another 24 h before casting.

### 2.2. Methods

#### 2.2.1. Open Porosity

After mixing the compositions according to the European Standard EN 196-1:1987 [47], six cylindrical samples with a diameter of 40 mm and 50 ± 5 mm high were cast and seal-cured at 20 ± 2 °C for 28 days. Afterwards, three of the samples were soaked in distilled water for 24 h until they reached equilibrium and then oven-dried at 105 °C for another 24 h until they reached equilibrium again. The three other samples were directly oven-dried at 105 °C for 24 h until they reached equilibrium. The methodology considered the conclusions proposed by Safiuddin and Hearn [48]. The addition of three only oven-dried samples was useful for estimating the salt leaching when the samples were soaked in water, especially for the high molarity compositions. The device used for the measurement of the mass change was a METTLER TOLEDO scale with a resolution of 0.001 g. Finally, the porosity *P* (%) was computed as follows:

(1) $$P=1-\frac{m_{\mathrm{dry}}/V_{\mathrm{dry}}}{m_{\mathrm{sat}}/V_{\mathrm{sat}}}$$

where $m_{dry}$ and $m_{sat}$ are the mass of the sample in dry and saturated conditions (g), respectively, and $V_{dry}$ and $V_{sat}$ are the volume of the sample in dry and saturated conditions, respectively. Moreover, $V_{dry}$ and $V_{sat}$ are considered the same, as the volume measurement did not show any significant difference. In all cases, the $m_{dry}$ is the dry mass obtained from the second set of samples that has not been soaked in water before drying, in order to avoid salt leaching.

#### 2.2.2. Water Vapour Desorption

The water vapour desorption samples were cast at the same time as the open porosity ones. Specifically, they were small cylinders with a diameter of 45 mm and a height of 5 ± 1 mm; the low thickness of the samples allowed them to reach equilibrium with the environmental conditions very fast and reduce the experimental time. Again, the samples were seal-cured for 28 days and then exposed to different relative humidity levels obtained through different saturated salt solutions at 20 °C. Table 3 shows the RH values and the corresponding salt solutions used to ensure them. Regarding the repeatability, three samples per RH level were cast and weighed regularly until they reached equilibrium. The device used for weighting the samples was a METTLER TOLEDO Columbus, Ohio (USA) scale with a resolution of 0.001 g. The samples were considered in equilibrium with the environment when the mass variation was lower than 1% over a two-week period. All the samples reached equilibrium within the first 28 days of exposure.

The final results were then used to compute the degree of saturation ( DoS ) at different relative humidity levels with the following equation:

(2) $$DoS_{\mathrm{RH}}=\frac{m_{\mathrm{RH}}-m_{\mathrm{dry}}}{m_{\mathrm{sat}}-m_{\mathrm{dry}}}$$

where $m_{\mathrm{RH}}$ is the mass at equilibrium at the target RH (g). Again, the dry mass is the one obtained for the samples not soaked in water before drying.

#### 2.2.3. BET Method

**Figure 1:**
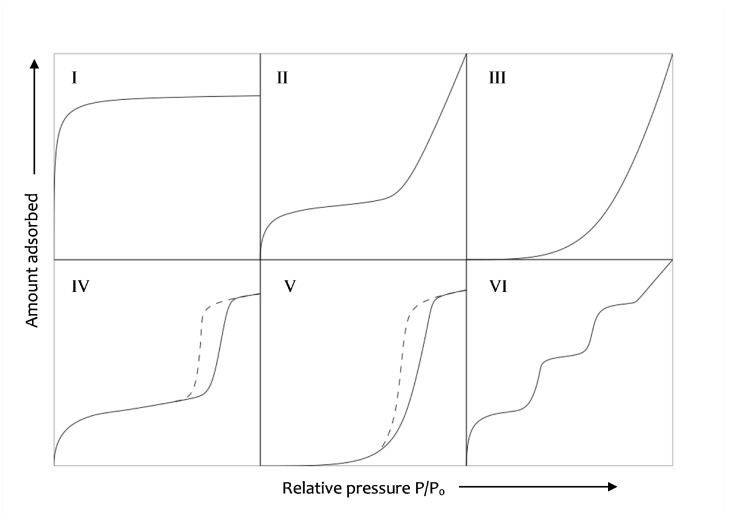
Desorption isotherm types.

The BET method [32,35,50] uses the data from the isotherm curves to compute the specific surface area of the pores. It is based on the Langmuir’s theory about the monolayer adsorption of gas molecules onto a solid surface. The assumptions are that the gas molecules will physically adsorb on a solid in layers infinitely, the different adsorption layers do not interact, and the theory can be applied to each layer. In order to apply this method, it is important that the monolayer and multilayer adsorptions have a clear transition; therefore, only isotherms II and IV from Figure 1 are suitable for this kind of computation.

The computation of the BET surface area needs a linear relationship between the relative pressure (or relative humidity) and the volume of adsorbed gas expressed as

(3) $$\frac{p/p_{0}}{X\cdot (1-p/p_{0})}$$

where *X* is the adsorbed volume ( $\frac{m^{3}}{g}$ ) and $\frac{p}{p_{0}}$ is the relative pressure. According to the model [34], experimental values corresponding to a relative pressure lower than 0.05 do not follow a linear trend, while between 0.35 and 0.5 there is a deviation from the straight line. As the use of saturated salt solutions does not allow for a very refined RH distribution, especially under 0.11, in our case, there were not three points within the range 0.05–0.35; nevertheless, we noticed that including the dried conditions and RH = 0.55 gives a linear relationship with the R^2^ always higher than 0.98; for this reason, we considered that it was still possible to apply the methodology, fully aware of the loss of accuracy in the final results. Finally, the BET specific area $A_{\mathrm{BET}}$ was computed as follows:

(4) $$A_{\mathrm{BET}}=\frac{X_{m}L_{\mathrm{av}}A_{m}}{M_{v}}$$

where $X_{m}$ is the monolayer capacity ( $\frac{m^{3}}{g}$ ), $L_{\mathrm{av}}$ is Avogadro’s number, $A_{m}$ is the cross-sectional area of the adsorbate equal to 0.108 nm^2^ for the water vapour [51], and $M_{v}$ is the molar volume (m^3^).

#### 2.2.4. BJH Method

The BJH method [30,33,39] allows us to compute the pore size distribution of a porous material, and it is based on the Kelvin–Laplace equation and Wheeler’s theory [40], a combination of BET multilayer adsorption and capillary condensation which can be summarised using the following equation:

(5) $$V_{s}-V=\pi \int_{r_{p_{n}}}^{\infty }{\left(r-t\right)}^{2}L\left(r\right)dr$$

where $V_{s}$ and *V* are the volume of adsorbed gas, respectively, at saturation and at pressure *p* (m^3^), L(r)dr is the length of pores with radius between *r* and r+dr (m), $r_{p_{n}}$ is the critical radius (m), and *t* is the multilayer thickness (m).

The computation followed the original model proposed by Barrett et al. in their paper [38], with a few modifications related to the use of water vapour instead of nitrogen. Specifically, the multilayer thickness $t(p/p_{0})$ was computed according to Badmann et al. in 1981 [52], as follows:

(6) $$t\left(p/p_{0}\right)=K_{1}+K_{2}\left(ln\left(-ln\left(p/p_{0}\right)\right)\right)$$

where $K_{1}$ and $K_{2}$ are constant coefficients that for water are equal to 3.85 and −1.89, respectively. The other difference is in the surface tension of the vapour, which we chose equal to that of the activating solution. In order to do so, we used the values provided by the Hazardous Substances Data Bank (HSDB) [53]; as they present a linear relationship between surface tension and NaOH concentration, it was possible to interpolate the data to match the exact concentration of the activating solutions. The change in the surface tension did not significantly affect the results of the computation, except for the 8 M compositions for which it allowed us to greatly reduce the difference between the BET surface area and the pore surface area obtained from the BJH model.

#### 2.2.5. Hydraulic Conductivity

The computation of the hydraulic conductivity follows the model proposed by Carette et al. [41], but without the local relative humidity data. Specifically, the model is based on the following nonlinear diffusion equation [41,54,55]:

(7) $$\frac{\partial S}{\partial t}=div\left(D_{w}\vec{grad}\left(S\right)\right)$$

where *S* is the degree of saturation and $D_{w}$ is the apparent coefficient of water diffusion ( $\frac{m^{2}}{s}$ ) which, in turn, can be expressed as

(8) $$D_{w}=K_{l}+D_{v}$$

where $K_{l}$ and $D_{v}$ are nonlinear coefficients describing the transport of liquid water and water vapour, respectively.

The liquid water flow can be expressed using Darcy’s law

(9) $$\vec{J_{l}}=-\rho_{l}\frac{k_{la}}{\mu_{l}}\vec{grad}\left(P_{l}\right)$$

where $J_{l}$ is the liquid water flux ( $\frac{\mathrm{kg}}{m^{2}\cdot s}$ ), $\rho_{l}$ is the water density ( $\frac{\mathrm{kg}}{m^{3}}$ ), $k_{la}$ is the apparent permeability to liquid water (m^2^), $\mu_{l}$ is the dynamic viscosity of water (Pa · s), and $P_{l}$ is the liquid water pressure (Pa).

(10) $$\vec{J_{v}}=-D_{va}\frac{M_{w}}{RT}\vec{grad}\left(P_{v}\right)$$

where $J_{v}$ is the water vapour flux ( $\frac{\mathrm{kg}}{m^{2}\cdot s}$ ), $D_{va}$ is the apparent diffusion coefficient of water vapour in air ( $\frac{m^{2}}{s}$ ), $M_{w}$ is the molecular mass of water ( $\frac{\mathrm{kg}}{\mathrm{mol}}$ ), *R* is the gas constant ( $\frac{J}{\mathrm{kg}\cdot \mathrm{mol}}$ ), *T* is the temperature (K), and $P_{v}$ is the partial vapour pressure (Pa).

The capillary pressure $P_{c}$ acting on the pores can be expressed as a function of the relative humidity, in agreement with Kelvin’s law

(11) $$P_{c}=\frac{-\rho_{l}RTln\left(h\right)}{M_{w}}$$

where $P_{c}$ is the capillary pressure (Pa) and *h* is the relative humidity.

The degree of saturation can be described using the van Genuchten equation

(12) $$S={\left(1+{\left(-a_{vg}ln\left(h\right)\right)}^{b_{vg}}\right)}^{-c_{vg}}$$

where $a_{vg}$ , $b_{vg}$ , and $c_{vg}$ are parameters obtained from the experimental results.

Combining Equations (7)–(11), it is possible to obtain the following equation to be solved for the moisture transport:

(13) $$\Phi \frac{\partial S}{\partial t}=\frac{1}{\rho_{l}}div\left(\vec{J_{l}}+\vec{J_{v}}\right)=div\left[\left(-\frac{k_{la}\rho_{l}RT}{\mu_{l}M_{w}h}-\frac{D_{va}M_{w}P_{v}^{sat}}{\rho_{l}RT}\right)\vec{grad}\left(h\right)\right]$$

where Φ is the material porosity, $D_{va}$ is the apparent diffusion coefficient of water vapour in air ( $\frac{m^{2}}{s}$ ), and $P_{v}^{sat}$ is the saturation water pressure (Pa).

In the last equation, the parameter $k_{la}$ depends on the degree of saturation, and it is the product of the intrinsic water permeability in saturated conditions $k_{l}$ (m^2^) and the relative permeability $k_{lr}$ . In addition, $k_{lr}$ can be determined using the Mualem conceptual relationship [41,56]

(14) $$k_{rl}\left(S\right)=S^{b_{mu}}{\left(1-{\left(1-S^{\frac{1}{a_{mu}}}\right)}^{a_{mu}}\right)}^{2}$$

where $a_{mu}$ and $b_{mu}$ are material parameters. In addition, $a_{mu}$ is considered equal to parameter $c_{vg}$ , while $b_{mu}$ is recommended to be between −4 and 5.5 for cementitious materials [41,57,58,59].

Similarly, the diffusion of water vapour is also dependent on the degree of saturation, according to the following Equation:

(15) $$D_{va}\left(S\right)=d_{rl}D_{0}$$

where $d_{rl}$ is the relative diffusion and $D_{0}$ is the diffusion coefficient of water vapour in the air. In addition, $d_{rl}$ can be expressed as

(16) $$d_{rl}\left(S\right)=\Phi^{a_{mq}}{\left(1-S\right)}^{b_{mq}}$$

where $a_{mq}$ and $b_{mq}$ are relative to the tortuosity of the material and therefore depend on the pore structure of the material. Their values range between 1.3 and 2.74 for $a_{mq}$ and 3.3 and 4.2 for $b_{mq}$ [41,60].

The computation allows us to identify the model parameters $K_{l}\left(S\right)$ and $D_{v}\left(S\right)$ for the relevant compositions and finally plot the predicted desorption isotherms using the van Genuchten model [41,61] and the hydraulic conductivity against the relative humidity. Specifically, the van Genuchten model takes into account the porosity and density of the porous material in order to provide a physical-based model of the material behaviour. The relevant compositions were the ones that did not present a mass increase and did not dry too fast. As a matter of fact, in some cases, the model did not converge, probably due to the use of it outside its limits of applicability. In addition, the model allowed us to identify the transport contribution of liquid water and water vapour on the hydraulic conductivity with respect to the relative humidity for all compositions.

It is important to highlight that the model itself computed the main parameters used to obtain the water vapour diffusivity and liquid water permeability of the material. Specifically, it computed $a_{vg}$ , $b_{vg}$ , $c_{vg}$ , $a_{mu}$ , $b_{mu}$ , $a_{mq}$ , and $b_{mq}$ .

##### Boundary Conditions

Considering the boundary conditions of the model, two factors were taken into account: the external relative humidity, which was considered equal to that of the saturated salt solution in the boxes, and a factor that takes into account the sample surface. This second factor considers an RH gradient in the air surrounding the sample surface through a “convection factor”. Specifically, it considers the speed at which the evaporated water from the sample surface is removed. It depends on the presence of convection inside the box, surface porosity, surface roughness, surface moisture, and temperature. This factor was calibrated experimentally by measuring the relative humidity at 2 mm from the surface of the sample [41]. For this experiment, it was been calibrated again, as it was assumed that the conditions between the two tests were similar enough not to cause a relevant difference. Moreover, a difference in the boundary condition factor would affect only the first days of drying when the flow of water from the material is strong and it does not significantly affect the results, even in the case of a significant variation.

## 3. Results and Discussion

### 3.1. Open Porosity

**Figure 2:**
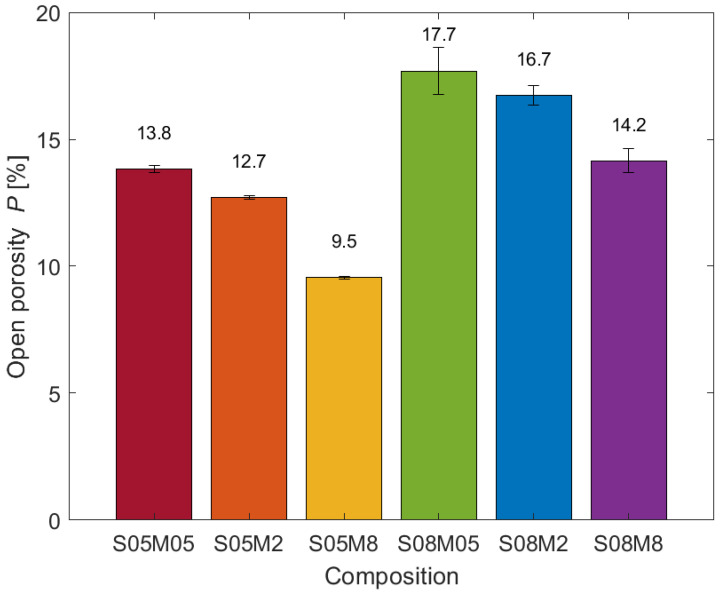
Open porosity.

Figure 2 shows the results of the open porosity test for all six compositions. The results present a distinct increase in the open porosity with the solution-to-binder ratio of the mix. This behaviour is in line with what has been observed when increasing the amount of water in PC mixtures [62]. Moreover, a decrease in the porosity was observed with increasing molarity. The results are then in line with previous studies on alkali-activated materials [25,63,64,65]. Specifically, the reduction in open porosity associated with the increased concentration of the activator is usually associated with a higher degree of dissolution of the precursor and a denser and more homogeneous microstructure [25,63,64].

### 3.2. Water Vapour Desorption

**Figure 3:**
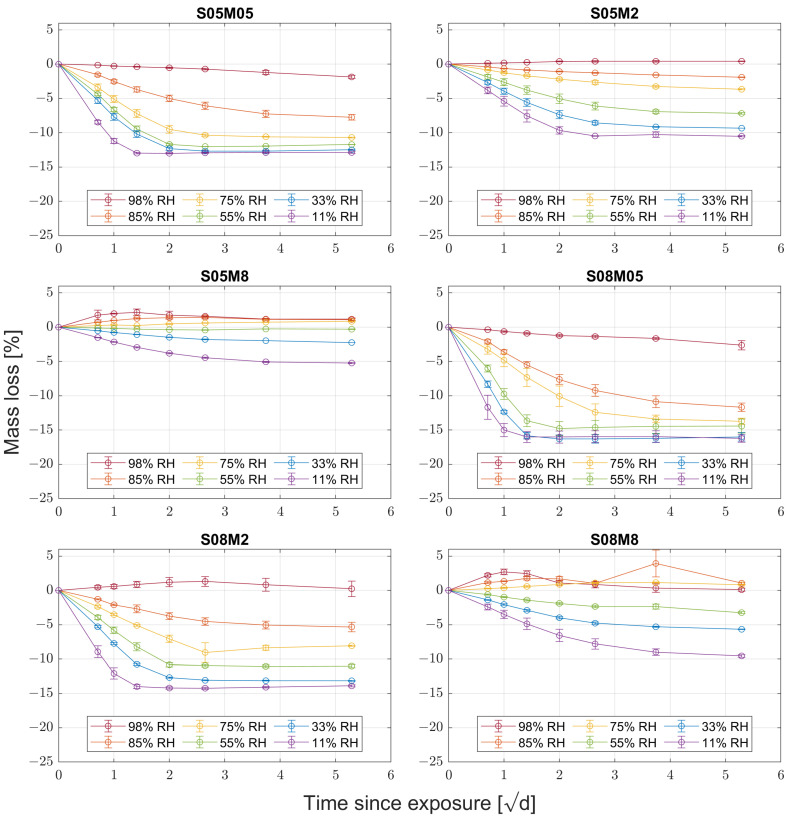
Mass loss over square root of time for the six different compositions.

Figure 3 shows the average mass loss for the six different compositions; the error bars represent the relative maximum and minimum for the different samples. From the results, it is possible to notice that increasing the molarity of the activator reduces the mass loss, regardless of the relative humidity. In addition, increasing the solution-to-binder ratio also increases the mass loss. Finally, 2 and 8 M compositions present a positive mass loss in high relative humidity conditions due to the initial relative humidity being lower than 98% for 2 M compositions and 75% for 8 M compositions.

**Figure 4:**
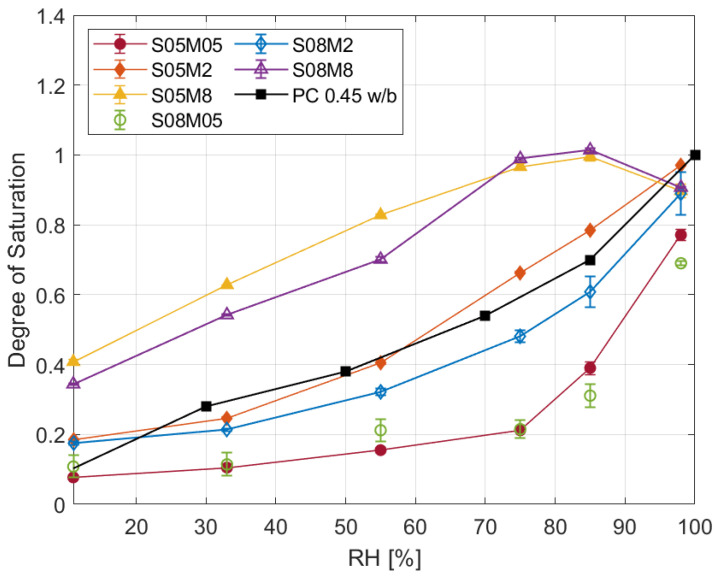
Degree of saturation at equilibrium for the six slag compositions and PC with w/b of 0.45 from [74].

The degree of saturation results for the different compositions are depicted in Figure 4.

The different curves can be coupled according to their molarity as they present similar behaviours, while the solution-to-binder ratio does not change the shape nor the position of the curves much. Moreover, increasing the molarity led to a higher degree of saturation, regardless of the relative humidity. On the other hand, it is very clear that the two 8 M compositions present an unusual behaviour, as the DoS at 98% RH is lower than the one at 85%. In order to verify the correctness of the experiment, a second set of samples has been tested, and it presented the same behaviour again; the cause of such results seems to be carbonation. The high molarity of the solution in the material causes an excess of weakly bounded Na_2_O [66,67] available for carbonation when it comes in contact with atmospheric CO_2_ [68,69,70], according to the following reaction:

(17) $$Na_{2}O+CO_{2}\to Na_{2}CO_{3}$$

**Figure 5:**
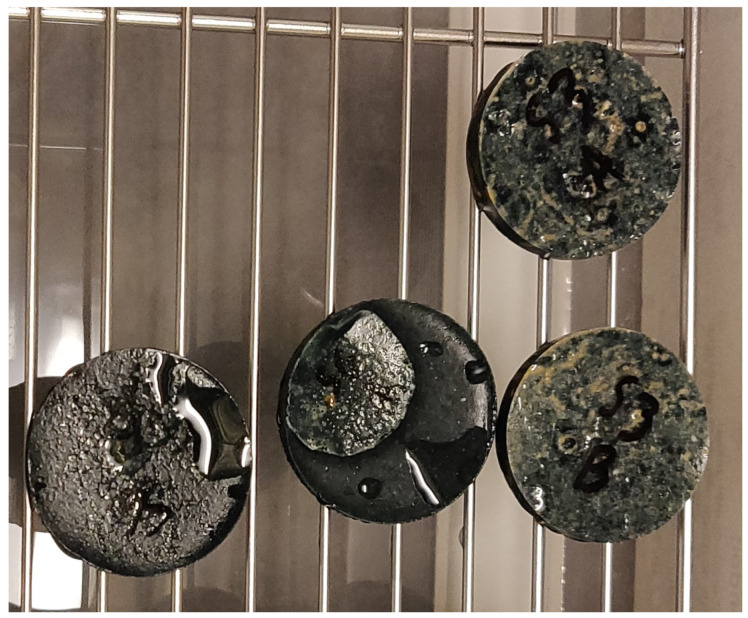
Samples for composition S05M8 and S08M8 exposed to 98% relative humidity.

The sodium carbonate formation consumes the sodium oxide, generating a concentration gradient between the inner part of the sample and its surface, forcing more Na_2_O to migrate towards the external layers of the material and fuelling the carbonation process even more. Finally, the presence of highly concentrated Na_2_CO_3_ causes deliquescence, a phenomenon for which the salt, instead of just locally changing the RH, liquefies and creates a saturated solution; the solution can then drip from the samples themselves and reduce their mass. According to the literature, deliquescence in sodium carbonate takes place when the relative humidity is higher than 75–76% [71], which agrees with what was observed during the test. As the samples exposed to 75% RH did not show signs of deliquescence, only the values for 85 and 98% RH were excluded from the following computation and modelling steps. Figure 5 shows a few samples for compositions S05M8 and S08M8 exposed to 98% RH with some visible liquid drops caused by the deliquescence process. According to the literature, the main effect of deliquescence is that it provides a liquid film on the surface of the material which, in turn, enhances the reaction between atmospheric gases and the material itself, leading to faster degradation and corrosion [72,73].

**Figure 6:**
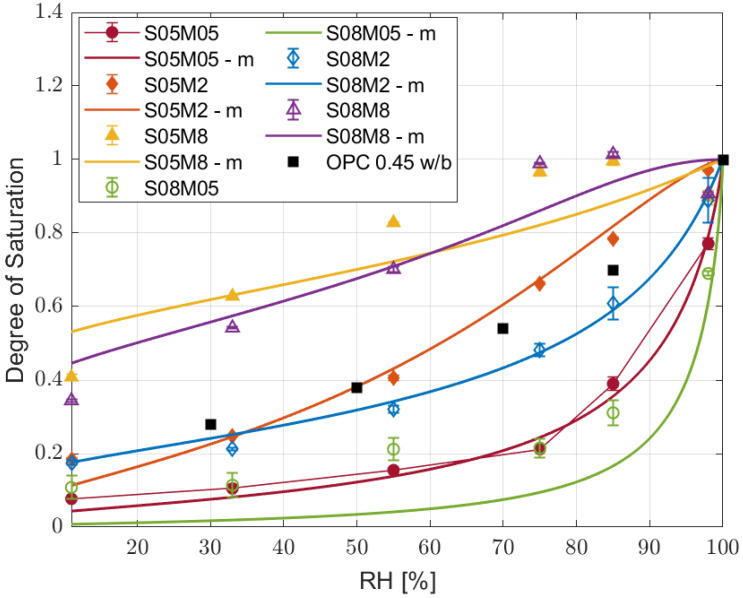
Modelled degree of saturation with respect to the relative humidity compared to the experimental one and to PC with w/b ratio of 0.45 from [74].

Figure 6 compares the experimental desorption isotherms with the computed ones.

The modelled curves were obtained from the hydraulic conductivity model. Based on the mass loss results of the drying experiments for all tested compositions, this model also identified the parameter values for the van Genuchten model for the desorption isotherms [30,52]. The modelled curves were therefore obtained not only from the identification of the experimental desorption isotherm results, but also from the evolution of the mass loss throughout the whole drying experiment of each composition. In addition, the model took into account the porosity and the density of the material. From a general point of view, the modelled curves present a good agreement with the experimental points, except for compositions S08M05 and S05M8. In the first case, even though the experimental results do not differ much from the ones obtained for S05M05, the change in density and porosity leads to very different modelled results. Moreover, the pore structure and the fast drying process made it impossible for the model to provide a better fitting of the data. The same is true also for composition S05M8. In addition, the 8 M compositions show a significant difference for the high relative humidity values as a result of the ineffectiveness of the van Genuchten model for such compositions. Finally, for all compositions, the model found it difficult to converge to the experimental data at 11% RH, as it is close to the limits of applicability of the model itself.

In addition, it is important to point out that the experimental results may have underestimated the water content of the material; specifically, carbonation has been proven to reduce the water content of Portland cement at every relative humidity level due to the clogging of the pores [75]. Even though specific studies on the topic for alkali-activated slag are still missing, it is possible that the same phenomenon occurs in our test as well. In order to verify the effects of carbonation on the water content of BFS, a second set of samples in noncarbonated conditions would be necessary. Nevertheless, as the duration of the test was very short, it is also possible that carbonation may be negligible, especially in environmental conditions.

As the computation of the desorption isotherm curves represented the first step in modelling the hydraulic conductivity of the alkali-activated mortars, their good or bad agreement with the experimental results indicates whether or not it was possible to proceed with the subsequent modelling steps. Table 4 summarises which compositions were considered suitable for further modelling, according to the agreement between the modelled and experimental degree of saturation shown in Figure 6.

### 3.3. BET Surface Area

**Figure 7:**
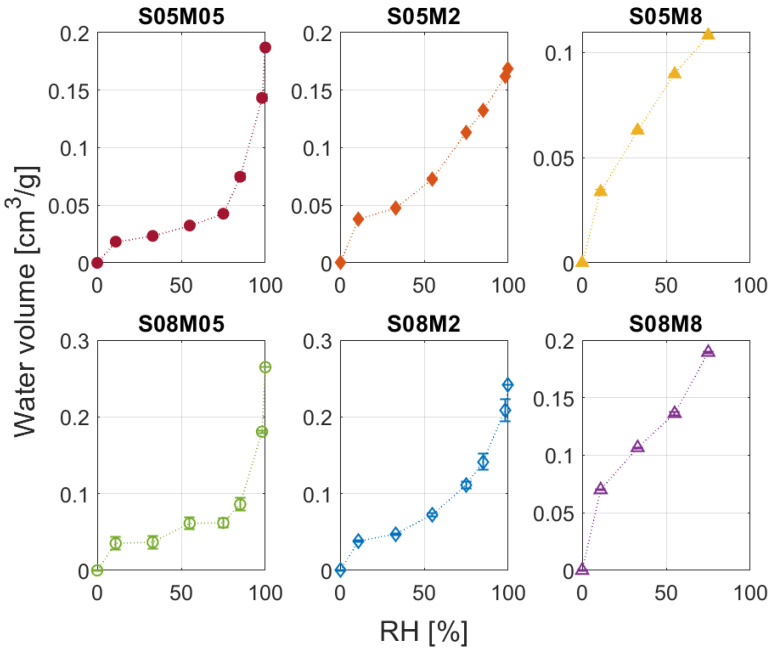
Water vapour desorption isotherms for the six compositions.

Before presenting in detail the results obtained from the computation, a brief comment on the isotherms obtained is necessary. Figure 7 shows the water volume against the relative humidity. All compositions present a type II isotherm except for S05M8 and S08M8, which show isotherms of types I and IV, respectively. For this reason, the analysis for S05M8 cannot be considered physically accurate, as its isotherm falls beyond the model applicability range. Nevertheless, as the methodology used was always the same, the computation can still provide a qualitative comparison with the other compositions.

**Figure 8:**
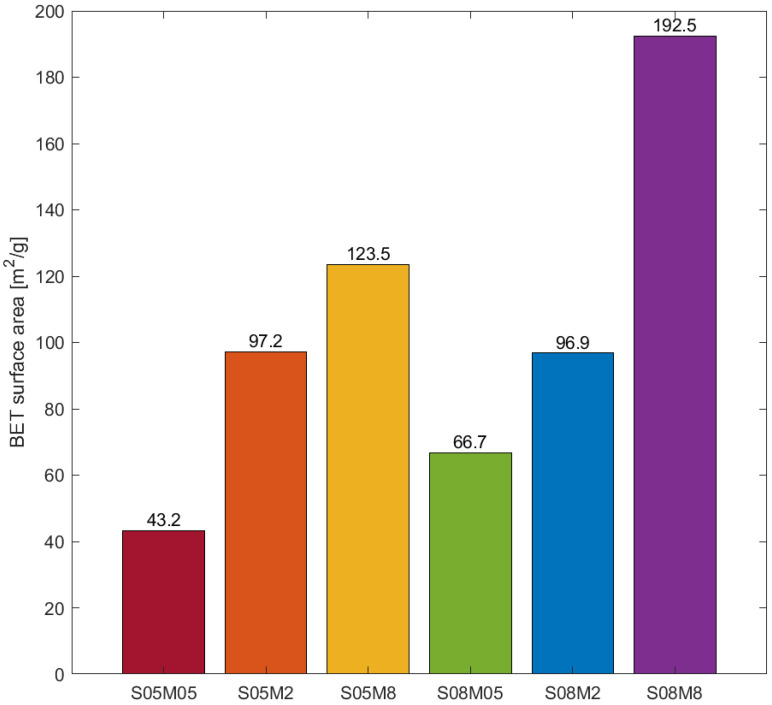
BET surface area.

The results of the BET specific surface area computation are summarised in Figure 8. Considering that the porosity decreases when increasing the molarity, the higher BET surface area was caused by a pore size refinement, which is in line with what was observed in the literature [21,25]. On the other hand, increasing the solution-to-binder ratio led to an increase in the surface area, which is due to the higher open porosity [25,76].

### 3.4. BJH Pore Size Distribution

**Figure 9:**
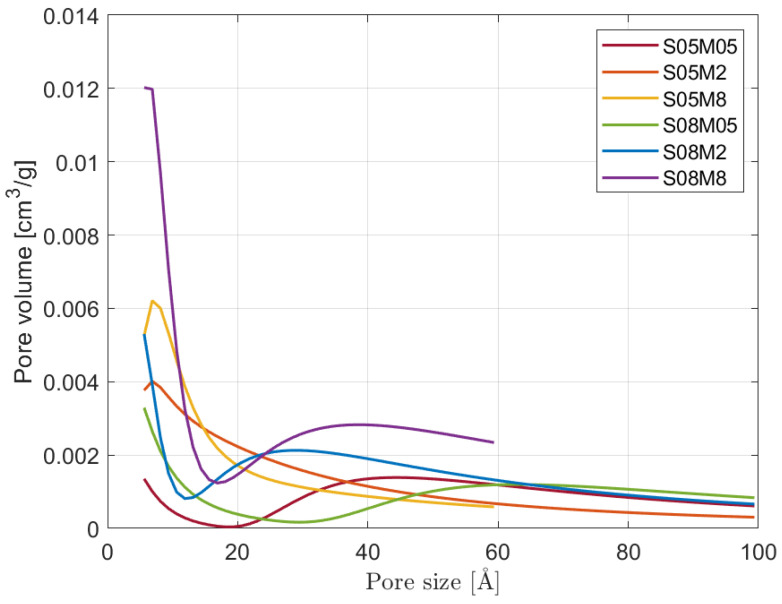
Pore size distribution of the six compositions.

**Figure 10:**
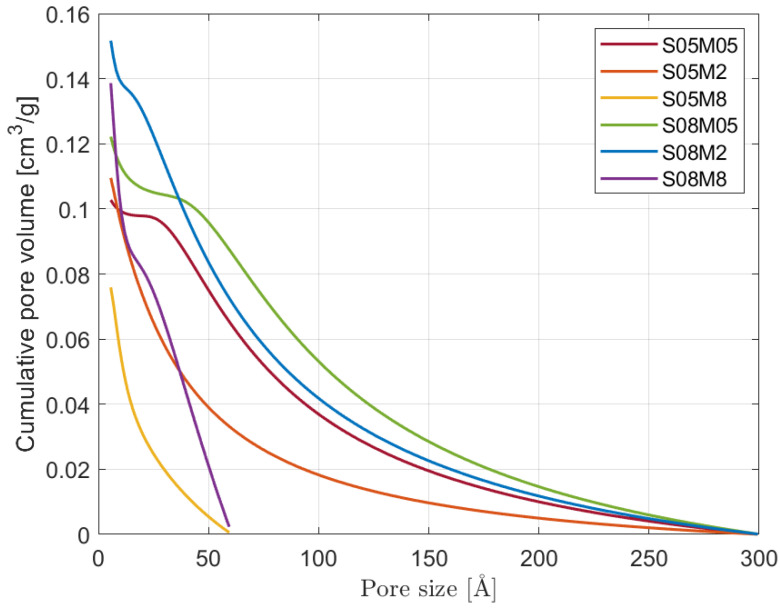
Cumulative pore size distribution of the six compositions.

The pore size distribution of the six compositions is shown in Figure 9, while the cumulative one is in Figure 10. All 0.5 and 2 M curves start from a maximum of 300 Å in order to cover the whole data set up to more or less 98% RH, while the curves for S05M8 and S08M8 begin at around 60 Å because it corresponds to 75% RH, considered as the upper limit for the experimental values, as explained in the previous section. The lower limit, on the other hand, is chosen equal to 5 Å, which corresponds to 11% RH, even though such small pores are difficult to investigate accurately with this kind of methodology. Nevertheless, it allowed us to use all the reliable experimental data and to have a good agreement between the BET surface area and the area of the pores obtained with the BJH method. Going back to the results, the difference in behaviour takes place mostly in the micropores range, that is, for pores smaller than 20 Å [11]:

- Note that 0.5 M compositions present a small number of micropores compared to the other compositions. The results are in line with what was observed for the porosity and the BET surface area: even though S08M05 presents the highest porosity, its surface area is relatively small due to a coarser pore structure, as visible in Figure 10.
- S08M05 does not present the highest open porosity value as it did during the open porosity test; a possible reason for this is that preliminary tests showed very low mechanical strength that may have allowed for a higher cracking formation during the drying phase in the oven, causing an increase in the number of connected pores and a subsequent higher mass loss. Specifically, the shrinkage experienced by the material due to the drying process leads to the appearance of tensile stresses that can cause the formation of microcracks, especially for materials with very low tensile strength [5]. As a consequence, the microcracks may increase the volume of open pores observed during the test [77,78]. S05M05 shows the same behaviour if compared to low s/b compositions.
- Note that 8 M compositions show a very steep increase in the pore volume in the micropore region, which is in line with the refinement of the pore structure observed in the literature [25,63], when increasing the molarity of the solution and the BET surface area results.

Even though the effects of carbonation on the pore structure are not clear, all compositions present a much finer pore structure than PC [50], even though the open porosity can be considered similar to PC [11].

Even though the increase in molarity was observed to increase both compressive strength and E-modulus [79], previous studies show that when the Na_2_O content is higher than 5.5% of slag, no significant increase in the strength development is observed [79,80,81]. On the contrary, increasing the Na_2_O content beyond 9% leads to efflorescence and brittleness [66,79,80].

In this case, as well, it is possible for carbonation to affect the final results: in PC, it was observed that carbonation reduces the open porosity and the pore size [75,82,83]. On the contrary, studies on alkali-activated slag show contradictory results. Puertas et al. [84] observed a refinement in the pore structure of the material with carbonation, while Ye and Radlińska [85] consider that the growth of crystalline products is strong enough to apply pressure on the pores’ walls, promoting the formation of microcracks. Finally, Humad et al. [86] observed an increased porosity in the carbonated area of the samples. As a consequence, it is difficult to understand the effect of carbonation on the pore size distribution of AAS, and further studies on noncarbonated samples are necessary.

Moreover, it is important to highlight the effect of the solution-to-binder ratio on the pore size distribution of alkali-activated slag. Specifically, from the results, it is possible to notice that increasing the s/b affects the pore size distribution differently according to the solution’s molarity: for the 0.5 M composition, it increases the number of pores smaller than 20 Å, for the 2 M compositions, it increases the number of pores bigger than 25 Å, and for the 8 M compositions, it increases the number of pores bigger than 20 Å and smaller than 10 Å. Alongside the change in the pore size distribution, the increase in s/b was observed to reduce the compressive strength and the E-modulus of the material [79].

### 3.5. Hydraulic Conductivity

**Figure 11:**
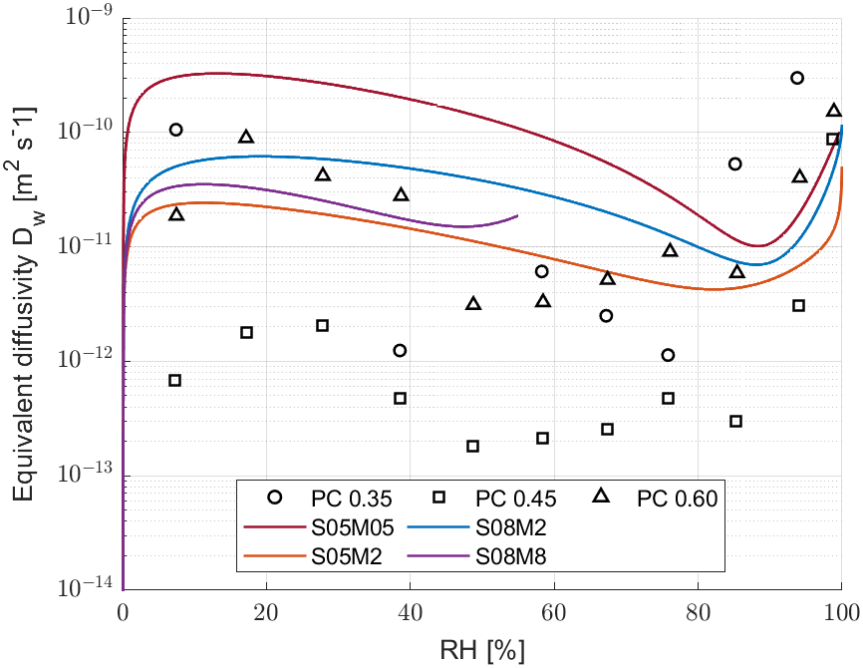
Equivalent diffusivity with respect to the relative humidity for the four compositions suitable for modelling compared to PC [87].

Figure 11 shows the evolution of the hydraulic conductivity with respect to the relative humidity.

**Figure 12:**
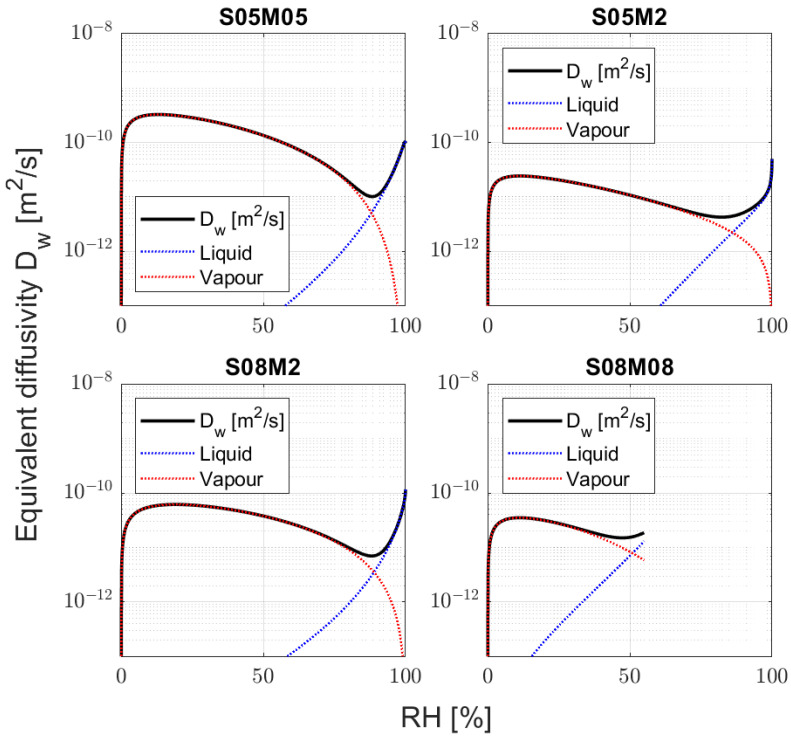
Liquid water and water vapour contribution to the hydraulic conductivity as a function of the relative humidity for the four compositions that present a good agreement between the model and the experimental values.

From a general point of view, the alkali-activated slag compositions present different behaviours according to their molarity. Specifically, S05M05 presents higher hydraulic conductivity regardless of the relative humidity compared to the 2 M ones and S08M8. In addition, the 0.5 and 2 M compositions present a minimum in the hydraulic conductivity, around 85% RH. In order to properly understand the hydraulic conductivity results of AAS, it is crucial to compare them with what has already been observed for PC. Specifically, Zhang et al. [87] report the hydraulic conductivity of PC against the relative humidity for three different cement pastes with w/b ratios of 0.35, 0.45, and 0.60. For the sake of clarity, the authors’ results are reported in Figure 11, as well. From the comparison emerges that S05M05 presents a higher hydraulic conductivity when compared to PC regardless of the RH. The 2 M compositions and S08M8 present values similar to PC 0.35 and 0.60 between 10% and 40% RH, a higher hydraulic conductivity between 50% and 80% RH, and finally a lower one between 90% and 100% relative humidity. Compared to PC 0.45, 2 M compositions present a higher hydraulic conductivity regardless of the RH. In addition, PC presents a minimum between 60% and 70% RH depending on the water-to-binder ratio, but in any case different from what was observed for AAS. The results are then in agreement with the high porosity and the fast drying process. Going into more detail about the results, Figure 12 shows the contribution of liquid water and water vapour transport as a function of the relative humidity on the final permeability for the four modelled compositions.

The results are interesting, as they show a clear difference with traditional PC. Specifically, we know that for PC the hydraulic conductivity drops when the relative humidity goes from 100 to 50%, slowing the drying process itself [41,88]. In addition, the water vapour transport is the main conductivity mechanism only when the RH is lower than 50% [41]. In the case of AAS, the hydraulic conductivity decreases from 100 to 85%, and then it increases again by a factor between 5 and 10 until the relative humidity reaches 50%. As a consequence, reducing the internal relative humidity speeds up the drying process itself, making the material more prone to cracking. From a practical perspective, having a minimum in the hydraulic conductivity at 85% RH means that AAS in in situ conditions, in which the relative humidity varies between 30% and 80% [89], dries much faster than Portland cement and is therefore more likely to present the formation of microcracks. Moreover, water vapour transport is the main mechanism in the drying process of alkali-activated slag not only at low RH levels. Finally, the methodology proved to be ineffective for 8 M compositions due to their swelling in high relative humidity conditions and the too-fast drying process at low RH values, which may also be an indicator of cracking in the specimens [90,91].

From the comparison of the different compositions, it is possible to notice that S05M05 and S08M2 present similar equivalent diffusivity at 100% RH, while S05M2 presents a lower one. The results seem to be in line with what has been observed in the previous sections about the open porosity and the pore size distribution of the different compositions. Specifically, a higher open porosity increases the equivalent diffusivity of the material due to the higher volume of connected pores, as observed in S08M2 [92]. At the same time, reducing the size of said pores reduces the equivalent diffusivity, as it makes it more difficult for the water to leave the material [92]. In this case, increasing the molarity reduces the pore size distribution and reduces the equivalent diffusivity, as observed for S05M2, while increasing the total number of pores increases it, as observed for S08M2.

In this case, as well, carbonation may have led to an underestimation of the hydraulic conductivity of the material, as in Portland cement, where a reduction in the conductivity and diffusion rates of water has been observed [93]. Once again, a comparison with noncarbonated samples is necessary to evaluate the effect of carbonation on the hydraulic properties of alkali-activated slag.

Concerning the modelled parameters of the material, Table 5 summarises the main results compared to the Portland cement ones [41]. In addition to the results, the table also presents the maximum and minimum values implemented in the model and obtained from [41].

From a general perspective, the values observed for alkali-activated slag are in line with what is to be expected for cementitious materials. The only one worth noting is $k_{l}$ , which represents the intrinsic water permeability of the material that does not present high accuracy. Specifically, it presents a variation up to two orders of magnitude, but high variability was already observed for Portland cement when changing the geometry of the samples [41]. In this case, though, the variability is higher and it may be related to the low contribution of liquid water transport when compared to PC.

## 4. Conclusions

The use of water vapour has been able to properly characterise the pore structure of alkali-activated slag, confirming what has been observed in the literature and providing a better understanding of the different compositions studied. In addition, it provided a very interesting insight on the drying mechanism and permeability of AAS. The main results obtained from the study are the following:

- Increasing the molarity of the activating solution refines the pore structure of the material.
- Increasing the solution-to-binder ratio not only increases the total porosity but also seems to refine the pore structure itself.
- High molarity compositions are not suitable for tests in high relative humidity conditions where carbonation takes place, as deliquescence greatly affects the results obtained.
- The tests on the total porosity of 0.5 M compositions show a discrepancy, possibly due to the test conditions and the low mechanical strength.
- The model proved to be able to predict the desorption isotherms from short drying experiments for AAS as well.
- The leading drying mechanism of alkali-activated slag is water vapour transport, making it fundamentally different from that of PC.
- The kinetics of drying strongly depends on external relative humidity, especially between 85 and 50%, which is the typical external RH.
- The modelling of the hydraulic conductivity is not applicable to high molarity conditions due to mechanical and modelling reasons, but also for the chemical activity—namely, carbonation and deliquescence—which fall out of the applicability of the model used, as it considers a fixed and unreactive microstructure.

## Figures and Tables

**Table 1:** Oxide composition of the slag [46].

Oxide	CaO	SiO_2_	Al_2_O_3_	MgO	SO_3_	TiO_2_	Fe_2_O_3_	K_2_O	Na_2_O	MnO	BaO
**Content [%]**	40.8	33.3	12.3	7.8	2.3	1.3	0.4	0.7	0.4	0.4	0.3

**Table 2:** Mortar mix proportions.

Composition	Solution-to-Binder Ratio	NaOH Concentration [M]	Sand-to-Paste Ratio	Water-to-Binder Ratio
S05M05	0.5	0.5	1	0.49
S05M2		2		0.44
S05M8		8		0.29
S08M05	0.8	0.5		0.77
S08M2		2		0.69
S08M8		8		0.43

**Table 3:** Saturated salt solutions [49].

RH	98%	85%	75%	55%	33%	11%
**Salt**	K_2_SO_4_	KCl	NaCl	Mg(NO_3_)_2_	MgCl_2_	LiCl

**Table 4:** Mortar suitability for further modelling of the hydraulic conductivity.

S05M05	S05M2	S05M8	S08M05	S08M2	S08M8
Yes	Yes	No	No	Yes	Only between 11 and 55% RH

**Table 5:** Values of the modelled parameters compared to Portland cement [41].

Parameter	S05M05	S05M2	S05M8	S08M05	S08M2	S08M8	PC	Min	Max
$k_{l}$	1.94 × 10^−21^	1.11 × 10^−23^	3.23 × 10^−22^	3.57 × 10^−22^	7.22 × 10^−22^	2.31 × 10^−20^	4.68 × 10^−22^	-	-
$a_{vg}$	10.23	1.37	1.05	0.82	1.03	1.95	1.65	-	-
$b_{vg}$	0.91	1.37	1.05	0.82	1.03	1.95	1.65	-	-
$c_{vg}$	1.09	0.99	0.28	1.72	0.54	0.20	0.39	-	-
$a_{mu}=c_{vg}$	1.09	0.99	0.28	1.72	0.54	0.20	0.39	-	-
$b_{mu}$	4.11	5.00	5.00	1.62	4.68	5.00	−1.57	−4.00	5.00
$a_{mq}$	2.02	2.72	3.02	2.70	2.61	1.63	3.00	1.30	2.74
$b_{mq}$	3.48	0.86	4.88	4.29	1.38	2.81	3.72	3.30	4.20

## Data Availability

Data are contained within the article.

## Abbreviations

The following abbreviations are used in this manuscript:

AAS	alkali-activated slag
OPC	ordinary Portland cement
s/b	solution-to-binder ratio
RH	relative humidity
WVD	water vapour desorption
DoS	degree of saturation
BET	Brunauer–Emmett–Teller
BJH	Barret–Joyner–Halenda
HSDB	Hazardous Substances Data Bank
